# A geometric semantic model and Parts-of-Sense Inference annotation framework

**DOI:** 10.3389/frai.2025.1666074

**Published:** 2025-11-13

**Authors:** Kiran Pala, S. Shalu, Vasudevan Nedumpozhimana, Kamal Kumar Choudhary

**Affiliations:** 1 Department of Social Sciences, University of Eastern Finland, Kuopio, Finland; 2 Department of Humanities and Social Sciences, Indian Institute of Technology Ropar, Rupnagar, India; 3 ADAPT Research Centre, Trinity College Dublin, Dublin, Ireland

**Keywords:** geometric semantic model, cognitive semantics, semantic annotation, Parts-of-Sense-Inference (POSI), cross-linguistic semantics, fine-grained semantic tags, multidimensional meaning representation, language processing

## Abstract

We introduce a geometric semantic model designed to capture fine-grained semantic representations in a multidimensional space. Building on this model, we develop a novel annotation framework that facilitates detailed semantic analysis across languages. Central to our approach is a set of Parts-of-Sense Inference (POSI) tags: 135 interpretable four-letter codes that annotate subtle semantic attributes often overlooked by traditional models. To evaluate the cross-linguistic and cross-structural applicability of this framework, we annotate expressions in four typologically diverse languages. Our results demonstrate that the proposed model provides an interpretable, cognitively plausible approach to semantic representation and can serve as a robust tool for investigating language processing and meaning inference across linguistic contexts.

## 1 Introduction

How sense and meaning are shaped by experience has long been central to semantic research, particularly within linguistic, cognitive, and philosophical inquiry. Traditionally, semantic theory has debated whether meaning is best understood as a reflection of prototypical concepts, abstracted through repeated exposure, or as the product of compositional features learned through experience and encoded in language (Rosch, 1975; Carey, 2009). This debate touches on a deeper issue: Are linguistic meanings preserved in symbolic representations, or are they also grounded in somatic and experiential interactions with the world (Lakoff, 1987; Barsalou, 1999, 2008)?

Recent research in cognitive linguistics and embodied cognition increasingly emphasizes the importance of perceptual grounding in semantic understanding. The sensory experience - visual, tactile, auditory - plays a fundamental role in how an individual form conceptual categories and comprehend language (Glenberg and Kaschak, 2002; Gärdenfors, 2004). The smell of coffee or the glow of a sunrise are not merely experiential events; they help structure how we conceptualize related objects, events, and their linguistic referents. This experiential substrate informs the syntactic and semantic properties attributed to expressions across languages.

Languages often encode semantic properties through multiword expressions (MWEs), where meaning emerges not from individual words but from their inferential interplay between them. For example, the expression spill the beans demonstrates an idiomatic shift in which literal meanings yield to culturally learned experiential constructs: it signifies reveal a secret through embodied knowledge of spilling (accidental release) and beans (scattered objects), consistent with (Lakoff, 1987; Barsalou, 1999). Such expressions rely heavily on shared embodied knowledge and often resist modeling by traditional distributional or logic-based semantic frameworks (Nunberg et al., 1994; Jackendoff, 2002).

Given these challenges, our work is motivated by the need for a semantic annotation framework that can capture the dynamic and experiential dimensions of meaning across typologically diverse languages, where variation in syntactic structure often conceals deeper conceptual regularities. We propose a multidimensional approach grounded in a geometric model of meaning, where “geometric” denotes a structured, interpretable semantic space rather than opaque embeddings built from perceptual and cognitive dimensions. This model focuses on three primary semantic dimensions experiential, spatial, and temporal which we argue are foundational to meaning construction in typologically diverse languages.

To clarify these dimensions and their differences (noting potential overlaps, such as experiential aspects influencing spatial metaphors), we define them as follows:

Spatial dimension captures configuration, location, and distribution of entities or concepts, emphasizing physical or metaphorical positioning (e.g., shape, proximity, or orientation like “up” vs. “down”). This dimension draws from conceptual spaces where spatial topology structures meaning (Gärdenfors, 2004). Temporal dimension encompasses duration, sequence, and patterns over time, including progression, cyclicity, or frequency (e.g., “before/after” relations or ongoing events). It models dynamic aspects distinct from static space but often intersects with it in metaphors (Pala, 2023b; Arstila, 2016). Similarly, the experiential dimension reflects attributes grounded in bodily and sensory interactions, subdivided into functional (utility-based, e.g., an object's purpose), structural (organizational, e.g., composition), material (sensory features, e.g., texture or color), and qualitative (abstract/symbolic, e.g., emotional value or cultural associations). This is rooted in embodied cognition, where meaning emerges from perceptual simulations (Barsalou, 1999).

These dimensions interact to form multidimensional representations, accommodating semantic gradience. For example, in the metaphorical expression “rising tension,” the spatial dimension contributes upward movement (vertical configuration implying increase), the experiential dimension adds qualitative intensity (emotional or sensory strain from bodily tightness), and the temporal dimension conveys progression over time (escalation from low to high). This interplay, aligned with conceptual metaphor theory, describes how abstract experiences are structured through perceptual grounding (Lakoff, 1987). Each dimension reflects a network of conceptual relations that arise through perceptual engagement and are systematically encoded in language use (Evans, 2009).

To operationalize this framework, we introduce a set of POSI tags: 135 interpretable four-letter codes designed to annotate fine-grained semantic attributes within these dimensions. Unlike part-of-speech tagging or fixed ontological categories, POSI tags capture dynamic, context-activated semantic properties rooted in cognitive experience. This makes the framework particularly effective for annotating MWEs, whose meanings often defy conventional syntactic or lexical classification.

We evaluated our approach by annotating MWEs in four structurally and typologically diverse languages. This multilingual evaluation supports our hypothesis that perceptually grounded dimensions of meaning are both cross-linguistically robust and computationally tractable. The proposed model offers a nuanced geometric representation of meaning that accommodates semantic gradience, overlap, and context sensitivity.

By integrating formal semantic theory with embodied cognition and multilingual linguistic data, this work provides a novel toolset for semantic analysis and offers theoretical insights into how experience shapes linguistic representation. The following sections describe the design of the geometric model, the structure, and rationale behind the POSI tags, and our methodology for multilingual annotation.

## 2 Geometric model of experiences

The geometric model of experiences serves as a metaphorical framework to analyze the complexity of perception, interpretation, and navigation of subjective realities. By analogizing geometric principles to experiential dimensions, this model extends spatial and dimensional concepts to explore how individuals construct meaning across emotional, sensory, and cognitive domains. Semantic properties of objects or representations are structured into three dimensions - spatial, temporal, and experiential proposed by Pala (2023b). Experiential properties further decompose into functional, material, structural, and qualitative aspects, which manifest as perceptual modality-oriented expressions in linguistic discourse (Pala and Shalu, 2025). This formalization enables systematic analysis of how inferential processes synthesis sensory, cognitive, and symbolic information into coherent representations.

Experiential properties hold particular significance due to their grounding in bodily sensations, which mediate both real-world interactions and imagined scenarios (Pala, 2023a). For example, experiencing a red apple involves integrating visual (redness), tactile (material texture), gustatory (sweetness or sourness), olfactory (smell), and cognitive (shape, temperature) inputs. Such multimodal correlations underpin the formation of representational content, as the synthesis of these properties shapes interpretable meaning. In figurative language, subjective experience drives inferential processes. The expression “John has a heart of gold,” for example, invokes qualitative attributes of gold (warmth, value) alongside spatiotemporal cues (“has a heart of”). This maps to the paraphrase “John is kind and thoughtful,” demonstrating how experiential properties guide metaphorical reasoning. To transition from conceptual framing to formal implementation, we introduce a structured semantic space that mirrors how experiential, spatial, and temporal features are encoded linguistically. This space underlies our POSI tagging system, where each four-letter code maps to salient coordinates along these dimensions. The term “geometric” is retained over “vector representations” to emphasize the structured, interpretable nature of this representational space. While grounded in ℝ^*n*^ vectors, the model transcends numerical embedding by encoding dimensions with semantic salience. Spatial, temporal, and experiential properties are mapped to subspaces that preserve perceptual and cognitive relationships. This allows modeling not only proximity but also metaphorical projection and analogical reasoning-processes aligned with embodied cognition and conceptual metaphor theory. For example, expressions like “rising tension” reflect spatialized conceptualizations of experience. Structuring semantic spaces geometrically ensures alignment with reasoning patterns (e.g., blending, interpolation), bridging formal semantics and cognitive processes. The framework provides a foundation for semantic annotation and computational applications. By formally encoding experiential properties in geometric vectors, it enables both theoretical analysis and practical tools in computational linguistics and cognitive science, while maintaining cognitive plausibility through its emphasis on spatial organization and cross-modal integration.

### 2.1 Formal definition of geometric semantic model

**Figure 1:**
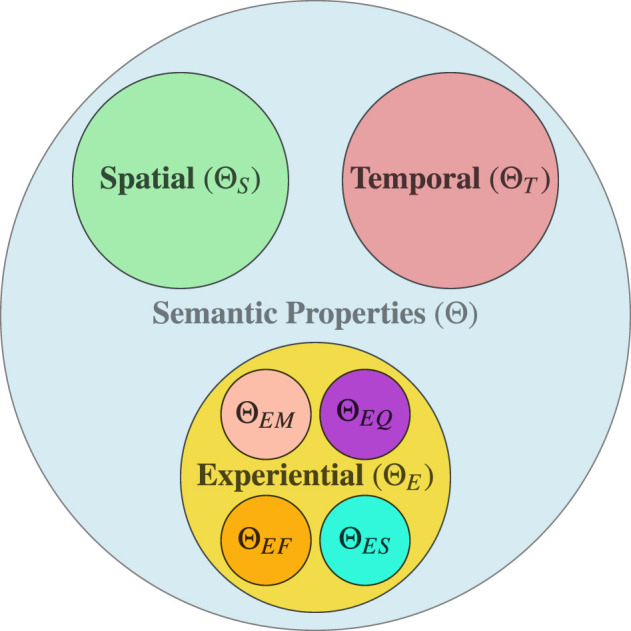
Abstract geometric semantic space (colour-coded for clarity).

Our geometric semantic model is a quintuple,


$$M_{g}=\left(L,\Theta ,\theta ,I,R\right)$$

where, L is the space of all linguistic expressions, Θ is the abstract geometric space, θ is the representational geometric space, I is the inference function, and R is the representation function. The space of linguistic expression (L) is a space of all syntactically valid linguistic expressions, which includes words, phrases, and sentences. The abstract geometric space is the space of spatial, temporal and experiential semantic properties, and its figurative illustration is shown in Figure 1. The abstract geometric space Θ is formally defined as:


$$\Theta =\Theta_{S}\times \Theta_{T}\times \Theta_{E}$$

where Θ_*S*_, Θ_*T*_, and Θ_*E*_ are the abstract geometric space of spatial (configuration, location, and distribution), temporal (duration, sequence, and temporal patterns), and experiential (sensory, functional, and symbolic attributes) properties of an entity or an object. The abstract geometric space of experiential property is further defined as:

$$\Theta_{E}=\Theta_{EF}\times \Theta_{ES}\times \Theta_{EM}\times \Theta_{EQ}$$

where Θ_*EF*_, Θ_*ES*_, Θ_*EM*_, and Θ_*EQ*_ are the abstract geometric space of functional (utility-based attributes), structural (organizational characteristics), material (sensory features), and qualitative (abstract/symbolic associations) subdivisions of experiential properties. This decomposition reflects the multimodal nature of experience, grounded in embodied cognition theory (Varela et al., 1991), where meaning emerges from sensorimotor interactions with the world.

Similar to the abstract geometric space, the representational geometric space θ is defined as:


$$\theta =\theta_{S}\times \theta_{T}\times \theta_{E}$$

and


$$\theta_{E}=\theta_{EF}\times \theta_{ES}\times \theta_{EM}\times \theta_{EQ}$$

where $\theta_{S}\in {\mathbb{R}}^{d_{s}}$, $\Theta_{T}\in {\mathbb{R}}^{d_{t}}$, $\theta_{EF}\in {\mathbb{R}}^{d_{ef}}$, $\theta_{ES}\in {\mathbb{R}}^{d_{es}}$, $\theta_{EM}\in {\mathbb{R}}^{d_{em}}$, and $\theta_{EQ}\in {\mathbb{R}}^{d_{eq}}$ are spaces of geometric vector representations corresponds to abstract geometric properties from Θ_*S*_, Θ_*T*_, Θ_*EF*_, Θ_*ES*_, Θ_*EM*_, and Θ_*EQ*_.

In this representational geometric space, spatial properties are represented as a real vector *d*_*s*_ dimensional consists of the feature values, such as the three coordinates, the shape and position of the object. The temporal properties are represented as a *d*_*t*_ dimensional real vector, which consists of features that capture the temporal dynamics of an entity or event. These features may include duration, frequency, temporal distance, cyclicity, or sequencing, each encoded as a real value. Each POSI tag corresponds to a specific projection or region in this representational geometric space, encoding fine-grained properties along one or more subdimensions (e.g., θ_*EM*_ for material texture, θ_*EQ*_ for symbolic resonance). This enables interpretable and structured semantic annotation grounded in the model's architecture.

The space of linguistic expression (L) is mapped to the abstract geometric space (Θ) through an inference function (I) and the abstract geometric space (Θ) is mapped to representational geometric space through a representational function (R).


I:L→Θ

R:Θ→θ

This framework models the interactions between subspaces of abstract geometric space. For example, the abstract geometric semantics of metaphor “*time flies”* is a combination of temporal (Θ_*T*_) and spatial (Θ_*S*_) meaning through experiential associations (Θ_*EQ*_). Such interactions can be modelled in representational geometric space through vector operations.

This model provides a bridge between embodied cognition theory and computational semantics. Mathematical formalisation of experiential dimensions enables systematic analysis of how language encodes complex experiences. Future work will explore dynamic weighting mechanisms for cross-cultural applications and integration with neuroimaging data for validation.

### 2.2 Comparison with other formal semantic models

The proposed geometric model (*M*_*g*_) assumes that meaning is derived from three core dimensions Spatial, Temporal, and Experiential. These dimensions are explicitly defined and multi-dimensional, enabling gradient representations of meaning. This framework offers a more holistic approach compared to formal semantics, which typically abstracts away sensory and symbolic details in favour of truth values. For example, in the metaphor “*time is a flowing river*”, the physical configuration of a river (directional flow, width, etc.) is captured by the abstract spatial property (Θ_*S*_) of this expression, and the progression of time as analogous to the flow of the river, is captured by the abstract temporal property (Θ_*T*_) of this expression. Then the utility of a river (e.g., transportation, sustenance) is captured by abstract experiential functional property (Θ_*EF*_); material properties like the fluidity of water relate to the continuous nature of time is captured by abstract experiential material property (Θ_*EM*_); and qualitative features like serenity or inevitability which enrich its metaphorical meaning is captured by abstract experiential qualitative property (Θ_*EQ*_) of the expression. These abstract properties will be represented in their corresponding representational geometric spaces. Where traditional formal models focus on logical validity or reference, the *M*_*g*_ model captures embodied, perceptual, and symbolic layers of meaning, offering interpretability and dimensional richness across linguistic phenomena.

A detailed comparison between traditional formal semantics primarily truth-conditional and model-theoretic approaches and our proposed geometric model (*M*_*g*_) is summarised in Table 1. This comparison highlights differences in focus, treatment of spatial, temporal, and experiential dimensions, representational granularity, and application scope.

**Table 1:** Comparison between formal semantics frameworks (truth-conditional and model-theoretic semantics) and the proposed geometric model (*M*_*g*_).

**Aspect**	**Formal semantics (truth-conditional & model-theoretic)**	***M*_*g*_ Model**
Focus	Truth values, possible worlds	Perceptual modalities, semantic interplay
Spatial & Temporal	Implicit in model structures	Explicit, encoded as θ_*S*_, θ_*T*_
Experiential Richness	Limited (e.g., cultural symbols not inherent)	Explicit (θ_*E*_ decomposition)
Application	Logical inference, compositionality	Perceptual grounding, metaphorical analysis
Granularity	High-level abstraction	Fine-grained, multi-dimensional

The geometric model explicitly encodes spatial, temporal, and experiential dimensions, offering a multi-dimensional, perceptually grounded approach to meaning representation.

#### 2.2.1 Truth-conditional semantics

In truth-conditional semantics, the meaning of a sentence is its truth value within a model:


M=(D,I)

where *D* is the domain of discourse, and *I* is an inference function mapping linguistic terms to elements of *D*. For example:


“X has a heart of gold”⊧“X is kind”.

In contrast, our geometric model (*M*_*g*_) decomposes both Θ and θ to capture the intricate interplay of sensory and symbolic meanings, which are underspecified in truth-conditional semantics. Our model offers a more nuanced understanding of metaphors and sensory experiences, which truth-conditional semantics overlooks. For example, in the metaphor “X has a heart of gold”, our model would integrate both the symbolic and experiential properties of gold, which truth-conditional semantics cannot fully capture.

This formalism supports compositional semantics, but typically leaves out perceptual or metaphorical meaning Hermann et al. (2013). Unlike binary truth assignments, *M*_*g*_ models the continuous, multimodal nature of metaphor, capturing affective nuance, material salience, and symbolic projection.

For example, the sentence “*the river flows westward*”, a truth-conditional analysis would evaluate whether the river's direction in the model corresponds to “westward”. However, it cannot account for the metaphorical richness of “Time is a flowing river” as experiential and symbolic elements are not encoded.

#### 2.2.2 Model-theoretic semantics

Model-theoretic semantics (Montague, 1970; Partee and Hendriks, 1997; Lewis, 1976)uses structures *M* = (*W, T, V*) where *W* is a set of possible worlds, *T* a temporal structure, and *V* a valuation function. This approach handles temporal and spatial dimensions, however lacks direct integration of experiential properties.

While model-theoretic semantics accounts for when and where events occur, it cannot explain how they feel, look, or symbolically resonate, elements that are core to cognitive interpretation (Lakoff and Johnson, 2008; Barsalou, 1999).

In contrast, our model explicitly incorporates experiential dimensions via Θ_*E*_ and its subcomponents (Θ_*EF*_, Θ_*EM*_, Θ_*ES*_, Θ_*EQ*_), and further grounds them in representational geometric spaces. For example, in our framework, the statement above is not only localized in time and space, but also enriched with symbolic, functional, and material properties of the river, such as flow dynamics (material), cultural symbolism (qualitative), and practical use (functional). This provides a more holistic model of meaning that integrates formal semantics with insights from embodied cognition and perceptual semantics.

For example, in the model-theoretic semantics, the statement “*the river will flow westward tomorrow*” is represented with a spatiotemporal structure *T, W*, allowing representation of future events. While model-theoretic semantics allows the encoding of spatiotemporal facts, it falls short in incorporating the experiential depth (e.g., the emotional impact of the river metaphor).

#### 2.2.3 Vector-space semantics

Vector-space semantics represent meanings as high-dimensional vectors derived from co-occurrence data Piantadosi et al. (2024); Turney and Pantel (2010). Despite modeling analogies and similarity, these representations lack interpretability grounded in perceptual meaning. While *M*_*g*_ also uses vectors, it grounds each dimension spatial (θ_*S*_), temporal (θ_*T*_), experiential (θ_*E*_) in interpretable perceptual and functional properties, enhancing transparency and cognitive relevance. Unlike opaque statistical embeddings, *M*_*g*_ provides structured semantics amenable to annotation and reasoning over meaning properties. Thus, *M*_*g*_ bridges the divide between cognitive plausibility and computational tractability, offering structured, interpretable semantic representations across dimensions.

### 3 Parts-of-Sense Inference (POSI) tag annotation framework

In this section, we define the POSI tag set and a framework for annotating natural language texts with their spatial, temporal, and experiential properties, as defined in the previously described geometric model. We defined 135 four-letter POSI tags using the first letters and/or the most prominent subsequent sounds of each word in the category name (e.g., *VUAB* for the *Vertical Up Above* category and *PSRC* for the *Past Recent* category), and all of these tags are listed in Table 2. With the help of a mapping (e.g. embedding) from such categorical features to a real-valued vector, these POSI tags can be viewed as proxies for vectors in the representational geometric space (θ).

**Table 2:** Category-wise list of all POSI tags (Parts–of–Sense Inference).

**Category**	**POSI-tag**
Spatial	VUAB, VDBL, VUOV, VDUR, VUTP, VDBT, VUUP, VULW, VULT, VURT, HLRA, HRLA, HWDT, MCEN, RLBS, RLNX, RLAD, RLDS, RLFA, RLNR, RLBH, RLWN, RLFR, RLBK, RLIS, RLOS, CNSA, EVSA
Temporal	PSRC, PSDS, PSRM, PRRC, PRNR, PREX, FTNR, FTDS, PSPF, PSIF, PSPG, PRPF, PRIF, PRPG, FTPF, FTIF, FTPG
EF	FNPR, FNST, FNTR, FNPS, FNPT, FNEE, FPHC, FSHC, FNLC, FNSL, FNPY, FNIE, FNNR, FNCO, FNAB, FNCS
EQ	QTSM, QTRG, QTGT, QTSL, QTHD, QTDN, QTML, QOFL, QOFR, QOSC, QOHR, QOSK, QOSW, QORT, QOBR, QOFS, QTSW, QTSR, QTST, QTBT, QTUM, QPTP, QPTL, QPHZ, QPMK, QPDN, QSMG, QSML, QSMS, QCRD, QCBL, QCGR, QCYL, QCOR, QCPL, QCBK, QCPK, QCBR, QCWT, QNFP, QABP
EM	MTML, MTPL, MTEW, MNOG, MTAB
ES	STCY, STSP, STRC, STPR, STTR, STOV, STCO, STCR, STAC, STPY, STFL, STAB
Fulfiller	FISE, FRSE, FHSE, FLSE, FMSE, FSSE, FCSE, FIJE, FRJE, FHJE, FLJE, FMJE, FSJE, FLMR, FNRE, FSYM

The experiential property from the geometric model is one of the key aspects emphasized in this study. We define numerous categories under the Functional, Material, Structural, and Qualitative domains.

Experiential Functional (EF) categories are defined based on the various functions or purposes each object, tool, service, or system can serve in our daily lives. For example, *Function Communication* (*FNCO*) is a category defined to annotate an object or concept that serves the act of communication, and *Function Utility* (*FNPY*) is a category defined for annotating an object or concept that has a physiological function in a living organism.

Experiential Material (EM) categories are defined based on the primary composition and nature of the materials. For example, *Material Metal* (*MTML*) is defined to annotate metallic objects like *knife* and *Material Natural Organic* (*MNOG*) is defined to annotate natural organic objects like *cotton*. We have additionally created a *Material Abstract* (*MTAB*) category to use when the material of something is not very concrete, for example, “honour” in the sentence “A crown made of honour”.

Experiential Structural (ES) categories are defined based on the geometric shape and structures. For example, *Structural Cylindrical* (*STCY*) category is defined to annotate cylindrical objects like *bottle* and *Structural Spherical* (*STSP*) category is defined to annotate spherical objects like *ball*. For annotating objects with undefined structures, we defined a *Structural Abstract* (*STAB*) category.

Experiential Qualitative (EQ) categories are defined based on different qualities such as texture [e.g., *Qualitative Texture Smooth* (*QTSM*)], odour [e.g., *Qualitative Odour Floral* (*QOFL*)], taste [e.g., *Qualitative Taste Sweet* (*QTSW*)], opacity [e.g., *Qualitative Opacity Dense* (*QPDN*)], and colour [e.g., *Qualitative Colour Red* (*QCRD*)]. Also, to denote the qualitative properties of concepts like love, hate, etc., we defined two more categories *Functional Qualitative Properties* (*FNQP*) and *Abstract Qualitative Properties* (*ABQP*).

The temporal properties indicate the time frame associated with an object, thing, or phenomenon and that can be past, present, or future. We defined 17 categories to mark information regarding temporal context. For example, we defined the *Present Near* (*PRNR*) category to indicate something occurred or is happening very close to the present time and *Past Distant* (*PSDS*) category to indicate something that occurred far in the past.

The spatial properties denote how objects, entities, or phenomena are positioned within a given space. Proposed POSI tags for the spatial axis are based on categorizing these positions across different dimensions and orientations. We proposed 28 categories to denote spatial properties. For example, we defined the *Vertical Up Above* (*VUAB*) category to denote an object is oriented vertically upward and *Medial Center* (*MCEN*) category to denote the object is positioned closer to the centre. Furthermore, we defined two categories: *Conceptual Spatial Abstraction* (*CNSA*) and *Eventive Spatial Abstraction* (*EVSA*) to denote events and entities whose spatial properties are ambiguous or unclear.

The essence of something may not be completely conveyed solely through semantic properties such as experiential, temporal, and spatial properties. Additional information or transitional complexities are essential for inferring the sense of representational content. For example, knowledge of the historical practice of soldiers biting bullets during surgeries without anesthesia during wars is required to understand the reason behind forming the idiomatic sense of the expression “*bite the bullet*”. To incorporate such additional aspects, the geometric model defined a new property called *fulfiller*. We defined 16 *fulfiller* categories to mark such external information. For example, we defined the *Historical Shared Experience* (*FHSE*) category to indicate the experience that took place in the distant past but is collectively remembered and often commemorated. If the *fulfiller* property is not defined or not required to infer the sense of representational content, we defined a *Not Required or Exist* (*FNRE*) category to indicate that.

## 4 Annotation

As an initial step, we manually analysed 100 English verb-noun idiomatic expressions selected from the VNC-Tokens dataset (Cook et al., 2008) and annotated with the proposed POSI tags. We focused on idiomatic expressions because we think linguistic expressions with figurative sense will be a suitable test case for validating the effectiveness of the geometric model and illustrating its nuances. Note that the geometric model used in this work posits that geometric properties of a figurative expression can be derived from the geometric properties of its constituent parts.

While we claim compositionality, we recognize that many figurative units are non-compositional: German “jmdm. einen Bären aufbinden” (“tell tall tales”), English “kick the bucket” (“die”) and Bangla “Ghorar dim” (“horse's egg” = “something impossible”) cannot be built from the spatial, material or functional properties of their parts alone. For such cases, the POSI fulfiller tags host the cultural/historical knowledge that bridges literal components and idiomatic sense, instead of forcing a geometric sum that never existed (Pala et al., 2025; DWDS, 2024; Nunberg et al., 1994).

We are not limiting the scope of our proposed model and annotation framework to idiomatic expressions and expressions from the English language. To validate the cross-structural applicability, we have annotated sets of transitive sentences and intransitive sentences from the British National Corpus (BNC) (Burnard, 2007; Consortium, 2007). To validate cross-lingual applicability, we selected three non-English languages, Punjabi and Bangla from the Indo-Aryan language family and Malayalam from the Dravidian language family. Then we have annotated five sample idiomatic expressions from each of these three non-English languages. We used GyanNidhi Multilingual Parallel Corpus and other linguistic resources in Indian languages (Consortium, 2008; Choudhary, 2019; Chaudhury et al., 2008; Arora et al., 2003; Gupta and Pala, 2012; Agrawal, 2008) for selecting expressions for the annotation. Annotated sample of an idiomatic expression, “*find foot*” is included in Appendix 1 and we will publicly release our entire annotated data.

### 4.1 Annotation process

To maintain the clarity and consistency of the annotation process, we followed specific guidelines:

- In the linguistic analysis of the corpus, three annotators with linguistic backgrounds were involved in the project. Additionally, for the Bangla and Punjabi languages, we consulted native speakers with linguistic backgrounds to verify annotations and gain a deeper understanding of the languages' context and nuances.
- To ensure reliability in our annotations, we adopted a majority vote approach throughout the process of analysing the data (Sabou et al., 2014; Davani et al., 2022). This procedure involved selecting the annotation with the highest level of agreement among the annotators at each point in time.
- In the initial step of the annotation process, the property relations for each component (words) in the idiomatic expressions were annotated with suitable tags. Following each tag, a description was given to explain or specify the tag used. Additionally, an order number was assigned to each property relation based on its significance in contributing to the meaning of the expressions.
- If a component had multiple potential POSI tags, the most specific tag was chosen as the most suitable. For instance, the structural properties of “moon” could be tagged as either STSP (*Structural Spherical*) or STAC (*Structural Arc*) or STCR (*Structural Circular*), but since STSP is more specific, it was selected as the most appropriate structural tag for “Moon”.
- In cases where assigning a specific tag to a component proves challenging, we applied the tag of the following word. For example, it is difficult to assign a function tag to the word "the." In such cases, we used the tag of the subsequent word. For instance, if the next word is “book,” we assign the function tag of the book which is “FNIE” to “the.”

### 4.2 Challenges

Similar to other studies focused on developing tag sets for annotating various types of data, we encountered numerous challenges. One of the primary issues we faced during the creation of the POSI tagset was the problem of sub-categorization. Each major category we used encompassed a set of subcategories, and designing subcategories that accounted for everything under the major categories proved to be a Herculean task. To achieve more precise and straightforward annotation, we opted for a more general classification linked to the major categories. For example, we categorized materials into polymers (e.g., bags, bottles, toys, etc.), metals (e.g., Gold, iron, copper, etc.), and natural materials (e.g., wood, wool, leather, etc.). Additionally, we developed a tagset (MTAB) to indicate the material of an object or expression when it is unclear or ambiguous. The same pattern was followed for all other major categories. The other challenge we encountered was the cross-linguistic applicability of the tag sets. Our study aims to create a set of tags that can be used across different languages. However, languages vary in how they express spatial and temporal relations. For instance, some languages use case suffixes or clitics to indicate spatial relations, while others use adpositions (e.g., Malayalam, Tamil, and Zulu). Additionally, some languages use morphological tense inflections on verbs to indicate time, whereas tenseless languages rely on adverbs, verbs with aspect and mood inflections, or other lexical items to establish time references (e.g., Chinese, Malay, Burmese, Thai). We have built the tagsets in such a way that they can account for different languages. The way language expresses the relations changes, but the fundamental relations associated with time and space remain the same in every language. Further, languages use different linguistic markers to indicate minute meaning changes (e.g., Polish, Hindi/Urdu, Malayalam, etc.). Addressing these elements in the tagset was one of our main concerns. To tackle this, we created a tagset called “linguistic markers-FLMR” and placed it under the fulfiller categories, as it accounts for the additional elements that contribute to meaning.

Another challenge we faced during annotation involved giving the proper temporal tag for the verbs that have the same base form, past form, and past participle form, such as “cut,” “hit,” and “put.” In these cases, we relied on contextual information or neighboring words to understand the forms and tagged accordingly.

## 5 Conclusion and future work

In this paper, we introduced a geometric semantic model that encodes meaning as interpretable vectors along spatial, temporal and experiential dimensions, together with POSI - 135 four-letter tags that annotate fine-grained, perceptually grounded semantic properties. A multilingual evaluation on English, Punjabi, Bangla, and Malayalam idioms, transitive/intransitive clauses and other MWEs showed that the scheme is cross-linguistically robust and cognitively plausible. By formalizing experiential, spatial, and temporal features that traditional models treat as peripheral, the framework bridges embodied-cognition insights and computational semantics, offering a unified account of how bodily experience, cultural knowledge, and linguistic structure jointly construct meaning, especially in non-compositional or metaphorical expressions.

Each POSI tag maps to a transparent region of the geometric space, so the resource can be plugged directly into downstream NLP pipelines: the tags supply explainable, embodied features to semantic parsers, metaphor detectors and machine-translation systems, while the fulfiller dimension supplies cultural or historical knowledge whenever compositionality fails.

In the future work, we will (i) automate POSI tagging via supervised learning, (ii) embed the tags into existing semantic knowledge bases, and (iii) extend the inventory to additional languages and genres. And we will undertake a quantitative evaluation-comparing POSI-augmented models against strong baselines on tasks such as semantic parsing to demonstrate the concrete effectiveness.

## Data Availability

The datasets presented in this study can be found in online repositories. The names of the repository/repositories and accession number(s) can be found in the article/supplementary material.

## B POSI-Tag Annotation of “*Find foot”* (Idiomatic and Literal Sense)

<Entry>
  <Text>Findfoot(Figurative)</Text>
  <Desc>To become comfortable in a new situation/
  environment/task or to begin to be confident or successful.</Desc>
<Spatial>
  <Tag>EVSA</Tag>
  <Desc>The spatial relation associated with the event indicated by the idiom is not clear or
    abstract</Desc>
<Order>-</Order>
</Spatial>
<Temporal>
  <Tag>PRRC</Tag>
  <Desc>A period close to the present.</Desc>
<Order>-</Order>
</Temporal>
<EF>
  <Tag>FNAB</Tag>
  <Desc>The function is not clear, but describe the
  process of be coming familiar with and
  effective in new situations.</Desc
>
  <Order>-</Order>
</EF>
<EQ>
  <Tag>QABP</Tag>
  <Desc>Confident, comfort, familiar, effective</Desc>
<Order>-</Order>
</EQ>
<ES>
  <Tag>STAB</Tag>
  <Desc>Strctural properties related to expression
    is abstarct</Desc>
<Order>-</Order>
</ES>
<EM>
<Tag>MTAB</Tag>
  <Desc>Material properties related to the expression
    is abstract.</Desc>
<Order>-</Order>
</EM>
<Word>
    <Text>Find</Text>
<Desc>To locate/ acquire something (verb)</Desc>
<Spatial>
<Tag>EVSA</Tag>
<Desc>The spatial relation associated with the
  verb ~find~ involves understanding the
    process of locating or discover ing an object
    , person, or place with in a given space</
    Desc>
<Order>5</Order>
</Spatial>
<Temporal>
    <Tag>PRRC</Tag>
    <Desc>A period close to the present</Desc>
    <Order>4</Order>
</Temporal>
<EF>
<Tag>FNAB</Tag>
    <Desc>Function is not clear, indicating
    achievement of something.  </Desc>
<Order>2</Order>
</EF>
<EQ>
    <Tag>QABP</Tag>
<Desc>Accuracy, Discovery, realization,
    Evidentiality, Exploration, Satisfaction</
    Desc>
<Order>1</Order>
</EQ>
<ES>
    <Tag>STAC</Tag>
    <Desc>ES of the following noun.</Desc>
<Order>6</Order>
</ES>
<EM>
    <Tag>MNOG</Tag>
<Desc>EM of the following noun.</Desc>
<Order>7</Order>
</EM>
</Word>
<Word>
<Text>Foot</Text>
<Desc>The lower extremity of the leg of humans and
    animals(countable noun)</Desc>
<Spatial>
    <Tag>VDBT</Tag>
<Desc>Something is positioned vertically and located at the lowest point or bottommost
        location.</Desc>
<Order>5</Order>
</Spatial>
<Temporal>
<Tag>PPRC</Tag>
<Desc>A period close to the present.</Desc>
<Order>4</Order>
</Temporal>
<EF>
  <Tag>FNPY</Tag>
<Desc>Support body weight and facilitate movement
    and balance</Desc>
<Order>1</Order>
</EF>
<EQ>
<Tag>QABP</Tag>
<Desc>Volitionality, flexibility, balance,
      adaptability, vulnerable</Desc>
<Order>1</Order>
</EQ>
<ES>
<Tag>STAC</Tag>
<Desc>Normal arch shape (flat foot/high arch foot/
    etc)</Desc>
<Order>6</Order>
</ES>
<EM>
<Tag>MNOG</Tag>
<Desc>Composed of various bones, muscles,
    ligaments</Desc>
<Order>7</Order>
</EM>
</Word>
</Entry>
